# Quantification and Improvement of the Dynamics of Human Serum Albumin and Glycated Human Serum Albumin with Astaxanthin/Astaxanthin-Metal Ion Complexes: Physico-Chemical and Computational Approaches

**DOI:** 10.3390/ijms23094771

**Published:** 2022-04-26

**Authors:** Syahputra Wibowo, Jessica Costa, Maria Camilla Baratto, Rebecca Pogni, Sri Widyarti, Akhmad Sabarudin, Koichi Matsuo, Sutiman Bambang Sumitro

**Affiliations:** 1 Department of Biology, Faculty of Mathematics and Natural Sciences, Brawijaya University, Jl. Veteran, Malang 65145, East Java, Indonesia; wibowo@student.ub.ac.id (S.W.); swid@ub.ac.id (S.W.); 2 Department of Biotechnology, Chemistry and Pharmacy, Università di Siena, Via A. Moro 2, 53100 Siena, Italy; jessica.costa2@unisi.it (J.C.); mariacamilla.baratto@unisi.it (M.C.B.); 3 CSGI, Consorzio per lo Sviluppo dei Sistemi a Grande Interfase, Via della Lastruccia 3, 50019 Sesto Fiorentino, Italy; 4 Department of Chemistry, Faculty of Mathematics and Natural Sciences, Brawijaya University, Jl. Veteran, Malang 65145, East Java, Indonesia; sabarjpn@ub.ac.id; 5 Hiroshima Synchrotron Radiation Center, Hiroshima University, Higashi-Hiroshima 739-0046, Japan; pika@hiroshima-u.ac.jp

**Keywords:** antioxidants, astaxanthin, astaxanthin metal complexes, human serum albumin, glycated human serum albumin, diabetes mellitus type 2, multi-techniques and computational analysis

## Abstract

Glycated human serum albumin (gHSA) undergoes conformational changes and unfolding events caused by free radicals. The glycation process results in a reduced ability of albumin to act as an endogenous scavenger and transporter protein in diabetes mellitus type 2 (T2DM) patients. Astaxanthin (ASX) in native form and complexed with metal ions (Cu^2+^ and Zn^2+^) has been shown to prevent gHSA from experiencing unfolding events. Furthermore, it improves protein stability of gHSA and human serum albumin (HSA) as it is shown through molecular dynamics studies. In this study, the ASX/ASX-metal ion complexes were reacted with both HSA/gHSA and analyzed with electronic paramagnetic resonance (EPR) spectroscopy, rheology and zeta sizer (particle size and zeta potential) analysis, circular dichroism (CD) spectroscopy and UV-Vis spectrophotometer measurements, as well as molecular electrostatic potential (MEP) and molecular docking calculations. The addition of metal ions to ASX improves its ability to act as an antioxidant and both ASX or ASX-metal ion complexes maintain HSA and gHSA stability while performing their functions.

## 1. Introduction

The accumulation of oxidative stress in biological systems causes a variety of health issues in humans [1]. Oxidative stress is caused by free radicals that exceed the defense capacity of human cells [2]. One of the oxidative effects generated by free radicals is a change in albumin protein structure in type 2 diabetic patients [3]. Albumin is a protein that is immediately influenced by an increase in blood free radicals. This problem impairs albumin’s ability to serve as a scavenger and transporter [4]. One of albumin’s functions, as a scavenger, is metal ion binding; as previously stated, heme possesses pro-oxidant qualities, and albumin is an effective protein in heme binding. When heme is linked with albumin, the pro-oxidant characteristics decrease, indicating that albumin functions as an antioxidant [5]. Albumin has also been shown to bind numerous metal ions such as Cu^2+^, Fe^2+^, Mn^2+^, Fe^3+^ and Mn^3+^ with active residues as Cys34 [6,7]. The oxidative stress target in albumin is site 2, and it is also shown that modification or removal of cysteine residue will expose human serum albumin (HSA) to be easily degraded [8]. Our previous studies showed that glycated human serum albumin (gHSA) had an unfolding event in molecular dynamic simulation within 15,000 ps. The unfolding also causes the detachment of side chains A and B in gHSA [9]. Changes in albumin’s secondary structure have an influence on blood rheology on a larger scale. One of the most important aspects of blood rheology is viscosity. Internal friction causes viscosity change, which is a measure of flow resistance. Blood viscosity is higher in diabetes mellitus (DM) patients due to red blood cell aggregation and impaired deformability. Microangiopathy and macroangiopathy are two types of blood vessel narrowing caused by diabetes. Slower blood and erythrocyte flow result from a combination of capillary shrinkage and increased blood viscosity [10,11]. Antioxidants are required by the body of T2DM patients to counteract protein unfolding and preserve the stability of proteins damaged by free radicals [12]. Antioxidants play a critical function in electron balance under oxidative stress situations. This is because antioxidants may serve as both donors and acceptors of electrons [13]. ASX, one of the lipophilic terpene substances with a polyene chain, is one of the carotene group’s antioxidants with a strong potential as an antioxidant. Free radicals can be stabilized by this chain [14,15]. As a radical scavenger, ASX employs a number of reactions, including radical adduct, hydrogen atom transfer and single electron transfer [16]. ASX treatment in gHSA and HSA has previously been proven to maintain protein structure until the endpoint of molecular dynamics simulation. The finding is confirmed by data from the backbone root mean square deviation (RMSD), root mean square fluctuations (RMSF) and movie visualization [9]. ASX is a carotenoid which can interact with a wide range of systems. ASX has two adjacent oxygen atoms in the form of hydroxyl groups and a keto group on the cyclohexane ring. Due to the existence of this oxygen atoms, ASX molecules can coordinate metal ions. The structure of cyclohexane is similar to hydroxyl quinones, which have high biological activity, including the formation of chelation complexes with transition metal ions [2]. Density functional theory analysis and molecular dynamic simulations showed that ASX, in the complex form with transition metal ions such as Cu^2+^ and Zn^2+^, has greater antioxidant capacity than ASX in the not complexed form [17,18].

In this research, the capacity of ASX and ASX-metal ion complexed with gHSA and HSA acting as antioxidants at different doses has been investigated. There has never been any research on the management of diabetes mellitus from the standpoint of albumin and ASX, as well as ASX-metal ions. Furthermore, CD spectroscopy has been used for time dependent investigation to monitor changes in the secondary structure of HSA and gHSA following interaction with astaxanthin. New data are also shown for antioxidant activity determination by EPR spectroscopy, rheology measurements, UV-Vis spectrophotometer analysis, particle size and zeta potential studies, as well as in silico approach. The purpose of this research is to determine the ability of the ASX and ASX-metal ions in maintaining protein structure as well as reduce viscosity levels in both gHSA and HSA.

## 2. Results and Discussion

Diabetes Mellitus is characterized by a variation in albumin protein structure caused by oxidative stress [3]. In this context, the interaction between gHSA/HSA and a strong antioxidant compound as ASX, was evaluated. Furthermore, to increase the complex capacity and the antioxidant activity of astaxanthin, the addition of metal ions such as Cu^2+^ and Zn^2+^ was studied.

### 2.1. UV-Vis Analysis

**Figure 1:**
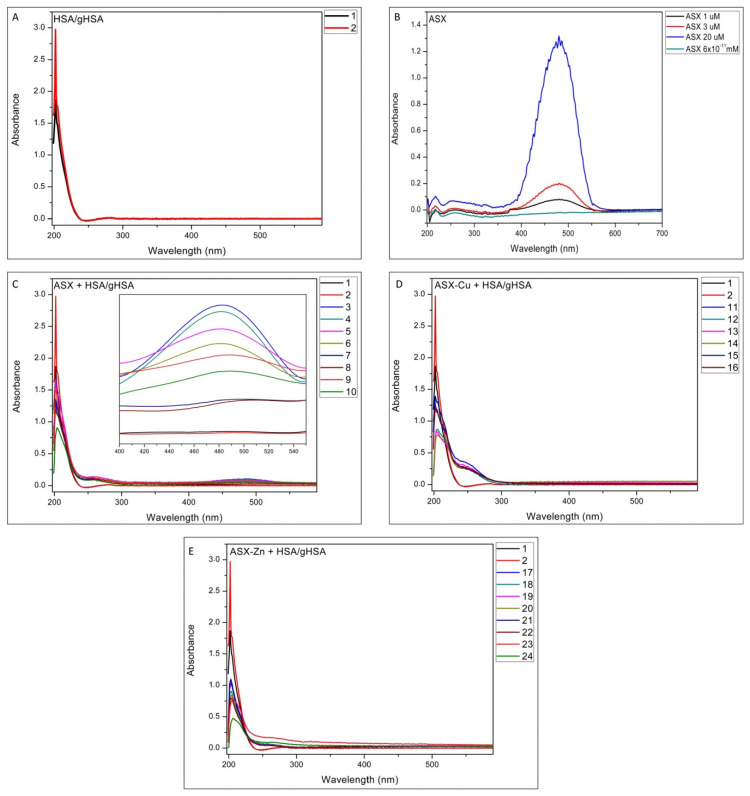
UV-Vis spectra of (**A**) HSA and gHSA; (**B**) ASX at different concentrations; (**C**) ASX complexed with HSA and gHSA using several molar ratios. The inset shows the absorbance peak variation in ASX in the range of 400–550 nm (**D**) ASX-Cu and HSA/gHSA complexes at different molar ratios and (**E**) ASX-Zn and HSA/gHSA complexes at different molar ratios. Sample codes are 1 (HSA 1 μM), 2 (gHSA 1 μM), 3 (gHSA-ASX 3 μM), 4 (HSA-ASX 3 μM), 5 (gHSA-ASX 20 μM), 6 (HSA-ASX 20 μM), 7 (gHSA-ASX 6 × 10^−11^ mM), 8 (HSA-ASX 6 × 10^−11^ mM), 9 (gHSA-ASX 1 μM), 10 (HSA-ASX 1 μM), 11 (gHSA-ASXCu (1 μM:1 μM)), 12 (HSA-ASXCu (1 μM:1 μM). 13 (gHSA-ASXCu (1 μM:2 μM), 14 (HSA-ASXCu (1 μM:2 μM), 15 (gHSA-ASXCu (3 μM:1 μM), 16 (HSA-ASXCu (3 μM:1 μM), 17 (gHSA-ASXZn (1 μM:1 μM), 18 (HSA-ASXZn (1 μM:1 μM), 19 (gHSA-ASXZn (1 μM:2 μM), 20 (HSA-ASXZn (1 μM:2 μM), 21 (gHSA-ASXZn (3 μM:1 μM), 22 (HSA-ASXZn (3 μM:1 μM), 23 (gHSA-ASXZn (20 μM:20 μM) and 24 (HSA-ASXZn (20 μM:20 μM).

In Figure 1A the UV-Vis spectra of HSA (black line) and gHSA (red line) are reported. Comparing the spectra of HSA and gHSA, an increase in absorbance was observed for the latter.

The increase in the absorbance peak of gHSA was attributed to the change in the secondary structure of the protein due to the formation of Amadori product occurring between glycation agents with the HSA lysines [19]. In Figure 1B, the UV-Vis spectra of ASX dissolved in EtOH at different concentrations, with the absorbance peak at 486 nm, is reported. Furthermore, in Figure 1C the UV-Vis spectra of HSA/gHSA-ASX complexes have been carried out. The presence of the peak at 486 nm is due to the presence of free astaxanthin which is not present in samples 7 and 8 (see the inset in Figure 1C). The UV-Vis analysis, performed in the presence of ASX and ASX metal complexes with HSA and gHSA, show the absence of free astaxanthin (see Figure 1D,E). This points out that the presence of metal ions favors a complete complexation of ASX with the protein. Furthermore, in all the spectra of HSA and gHSA complexed with ASX and ASX-metal ions, an absorbance decrease in the region of 200 nm was observed compared to the free forms, due to the conformational change of the protein. These results confirm the complexation of ASX and ASX-metal ions with HSA/gHSA. Previous studies on the bovine serum albumin (BSA) complex for reaction with different antioxidants reported similar results. A decrease in the absorbance peak, when the antioxidants were complexed with glycated albumin, was detected [20,21,22]. The decrease in absorbance values is also supported by CD data, where a change in the secondary structure of the protein was observed during incubation time.

### 2.2. EPR Analysis

EPR spectroscopy has been applied to investigate the Cu^2+^ complexation ability of ASX, gHSA-ASX-Cu and HSA-ASX-Cu as well as the antioxidant activity through the in vitro assay of 2,2-diphenyl-picrylhydrazyl (DPPH).

#### 2.2.1. Complexation Ability

**Figure 2:**
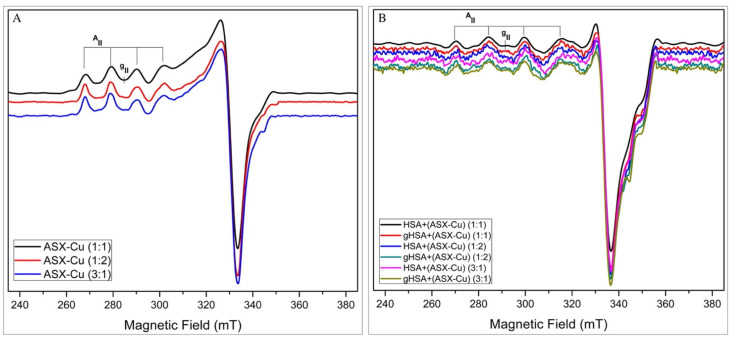
X-band EPR spectra of: ASX-Cu 1:1 (black line), ASX-Cu 1:2 (red line) and ASX-Cu 3:1 (blue line) (**A**). HSA+(ASX-Cu) in the molar ratios: 1:1 (black line), 1:2 (blue line), 3:1 (pink line) and gHSA+(ASX-Cu) in the molar ratios: 1:1 (red line), 1:2 (green line), 3:1 (brown line) (**B**). Experimental conditions: T = 150 K, ν = 9.67 GHz, 21 mW microwave power and 0.5 mT modulation amplitude.

In Figure 2A, low-temperature (150 K) EPR spectra of ASX-Cu in the 1:1, 1:2 and 3:1 molar ratios are reported. The figure shows the typical line shape of Cu^2+^-complexes in an axial geometry with defined parallel and perpendicular regions. These data confirm the ability of ASX to complex copper ion. From the analysis of the parallel region of the EPR spectra, the presence of the two copper isotopes is evident, due to the slight splitting of the single lines. For all the spectra, the inhomogeneous broadening of the lines and the experimental magnetic parameters, g_║_ = 2.425 ± 0.002 and A_║_ = 11.0 ± 0.2 mT, are typical for copper complexes with 4O atoms in the coordination sphere, as it is also confirmed by the Blumberg and Peisach diagram [22]. The assignment of Cu–O bonds in the EPR spectra is in agreement with a previous study on UV-Vis and FTIR of ASX-metal ions complex [18].

In Figure 2B, low-temperature (150 K) EPR spectra of HSA+ASX-Cu and gHSA+ASX-Cu in the molar ratios 1:1, 1:2 and 3:1 are reported. The figure shows the typical line shape of Cu^2+^-complexes in an axial geometry, but the presence of coordinated nitrogen atoms cannot be ruled out. It is evident from the broadening of the parallel components of the spectra compared with the ones reported in Figure 2A. The magnetic parameters for these spectra are g_║_ = 2.363 ± 0.002 and A_║_ = 15.4 ± 0.2 mT supporting a 1N3O coordination [23]. For comparison, the EPR spectra of Cu^2+^ with HSA and gHSA were recorded (Figure S1 red and blue lines respectively) showing the complexation ability of the protein. These EPR data clearly show that stable complexes can be formed by the interaction of ASX-Cu with HSA and gHSA at all the tested molar ratios. This result strongly supports the initial theory and results for CD analysis that the addition of ASX and ASX-metal ions, in this case Cu^2+^, can maintain the stability of HSA and gHSA proteins. The same findings, regarding the ability of albumin to bind Cu^2+^, were also investigated by Sciortino et al. [24]. In prion proteins (PrP^c^), complexes with Cu^2+^ are also found in the “octarepeat” regions and show the same pattern when Cu^2+^ binds to the N-terminal of the protein [25,26].

#### 2.2.2. Antioxidant Activity

**Figure 3:**
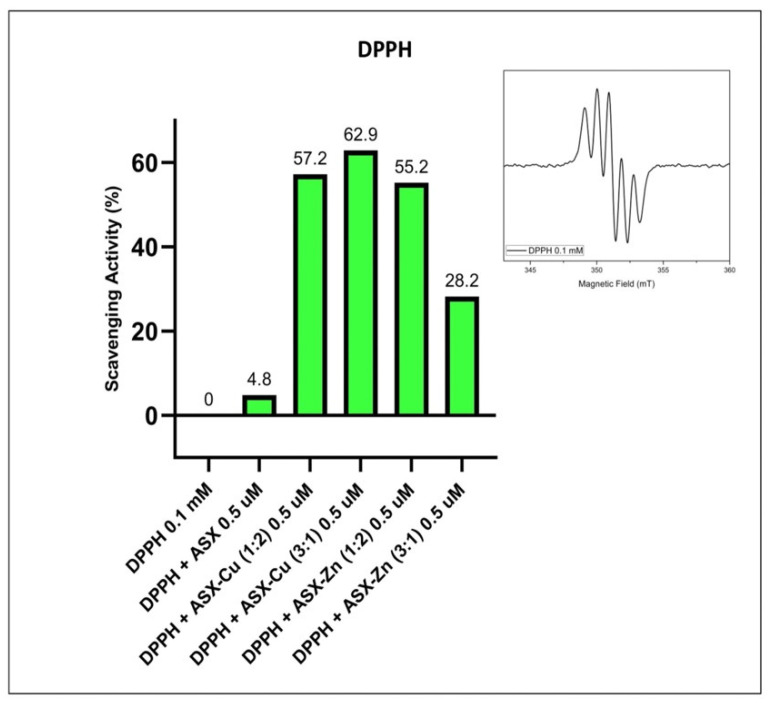
Scavenging activity percentage of ASX and ASX in the presence of metal ion Cu^2+^ and Zn^2+^ at different molar ratios. In the inset: room temperature EPR spectrum of the DPPH radical. EPR spectra were recorded at room temperature after 30 min of incubation. Experimental conditions: ν = 9.86 GHz, 2.1 mW microwave power and 0.1 mT modulation amplitude.

The antioxidant activity of ASX and ASX-metal ions was tested by EPR using the DPPH assay (Figure 3) [27]. In Figure 3 the percentage of the scavenging activity of ASX and ASX plus metal ions Cu^2+^ or Zn^2+^ at different molar ratios is reported. In the inset, the room temperature EPR spectrum of the DPPH radical is shown. It is characterised by five lines due to the hyperfine interaction of the unpaired electron with the two magnetically equivalent N atoms present in the structure.

The figure shows that at the same concentration of 0.5 μM, free astaxanthin has a scavenging activity of 4.8% compared to 62.9% of ASX-Cu complex (3:1) and 55.2% of ASX-Zn (1:2). It was found that the ratio (3:1) is the optimal concentration for ASX-Cu complex showing the higher percentage of scavenging activity when compared to the other ratios. This is in agreement with Marin et al. [28], who demonstrated that the addition of metal ions to ASX increased its antioxidant activity.

### 2.3. Molecular Electrostatic Potentials (MEP)

As a ketocarotenoid, ASX with the structural formula C_40_H_52_O_4_ has a molar mass of 596.84 g/mol [29]. This compound has two terminal rings joined by a polyene chain with either single or double bonds. There are various types of ASX, including the stereoisomeric form, esterified form (both hydroxyl groups react with fatty acids), geometric isomer and free form. ASX has two asymmetric carbon atoms in the 3,3 positions of the β-ionone ring as well as -OH (hydroxyl) functional groups on both ends of the molecule. Natural sources contain all of these forms, with the stereoisomeric forms (3S, 3′S) and (3R, 3′R) being the most prevalent [30]. Unlike other carotenoids, both rings are oxygenated, with polyene chains double-bound as a backbone, boosting ASX’s antioxidant capabilities [31].

**Figure 4:**
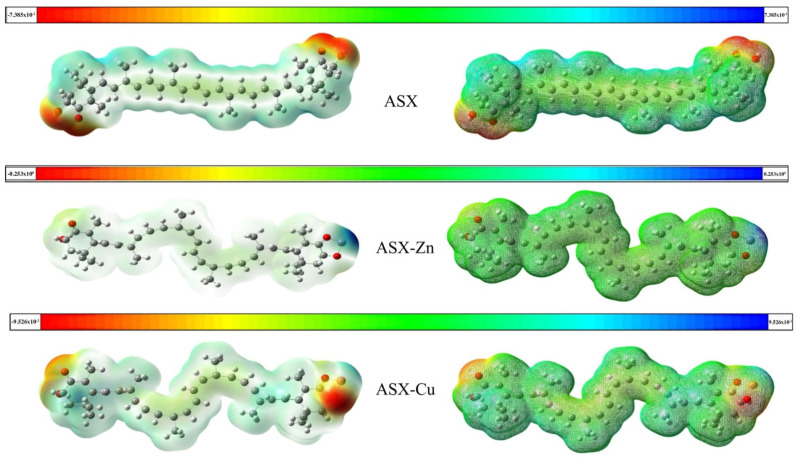
Graphical depiction of molecular electrostatic potentials of astaxanthin (**above**) and astaxanthin with Zn^2+^ (**center**) and Cu^2+^ (**below**). Variation in color distribution indicate different electrostatic properties (yellow: medium electron-rich site, blue: electron-deficient site, light green: almost neutral site, red: intense electron-rich site, white or grey: zero potential).

MEP is a critical metric for determining the reactivity of an approaching electrophile towards a compound active center (where the electron distribution effect is most prominent). MEP analysis is important because it represents molecular size structure and charges of a compound. The MEP data are highly valuable in the research of molecular structure and its physico-chemical property relationship. MEP analysis was carried out for astaxanthin and astaxanthin in the presence of Cu^2+^ and Zn^2+^. A change in electrostatic properties was observed. In Figure 4, the maximum negative region, which is the preferred site for the electrophilic attack, is indicated in red as the majority of MEP, whereas the maximum positive region, which is the major site for the nucleophilic attack, is indicated in blue. The energy range of astaxanthin was −7.385 × 10^−2^/7.385 × 10^−2^/characterized by nucleophilic regions (red zone) in correspondence of oxygens. In presence of Zn^2+^, the energy range of astaxanthin moves to −0.253 × 10^0^ /0.253 × 10^0^ due to the electrophilic regions (blue zone) near the zinc. Meanwhile, in presence of Cu^2+^ the nucleophilic regions are observed in correspondence of copper and the energy range of complexed astaxanthin increased to −9.526 × 10^−2^ /9.526 × 10^−2^. The alteration of astaxanthin structure in the presence of metal ions was also revealed in our previous study [17]. The transition from trans to cis conformation was observed in the complexes of astaxanthin with metal ions. This cis modification could improve astaxanthin’s antioxidant capabilities [32]. These results support the best scavenging activity shown by the ASX-metal ion complexes obtained with DPPH test by EPR spectroscopy.

### 2.4. CD-Spectroscopy

**Figure 5:**
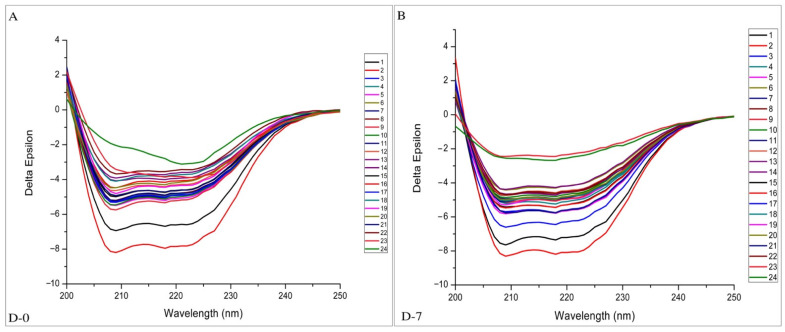
CD spectra of all samples (the numbers refer to the different samples reported in Materials and Methods) after incubation at T = 310 K recorded at day 0 (D-0) (**A**) and day 7 (D-7) (**B**).

**Figure 6:**
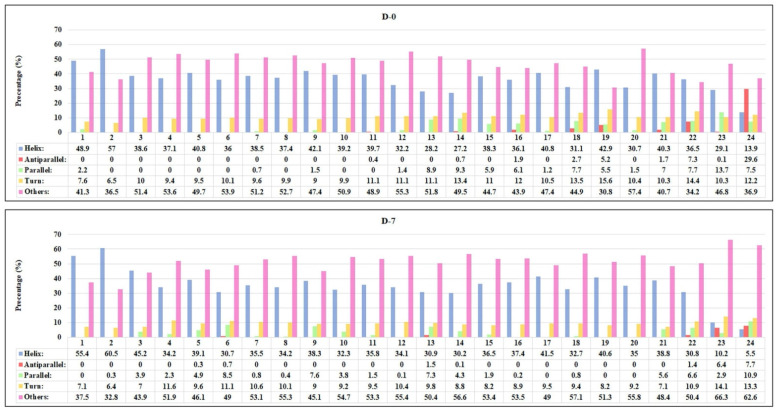
Percentage of secondary structures calculated using the BestSel program referred to D-0 and D-7: **1** (HSA 1 μM), **2** (gHSA 1 μM), **3** (gHSA-ASX 3 μM), **4** (HSA-ASX 3 μM), **5** (gHSA-ASX 20 μM), **6** (HSA-ASX 20 μM), **7** (gHSA-ASX 6 × 10^−11^ mM), **8** (HSA-ASX 6 × 10^−11^ mM), **9** (gHSA-ASX 1 μM), **10** (HSA-ASX 1 μM), **11** (gHSA-ASXCu (1:1) μM), **12** (HSA-ASXCu (1:1) μM), **13** (gHSA-ASXCu (1:2) μM), **14** (HSA-ASXCu (1:2) μM), **15** (gHSA-ASXCu (3:1) μM), **16** (HSA-ASXCu (3:1) μM), **17** (gHSA-ASXZn (1:1) μM), **18** (HSA-ASXZn (1:1) μM), **19** (gHSA-ASXZn (1:2) μM), **20** (HSA-ASXZn (1:2) μM), **21** (gHSA-ASXZn (3:1) μM), **22** (HSA-ASXZn (3:1) μM), **23** (gHSA-ASXZn (20:20) μM) and **24** (HSA-ASXZn (20:20) μM).

CD-spectroscopy was used to investigate the changes in the protein secondary structure of HSA and gHSA with or without ASX and ASX-metal ions in different molar ratios. Their stability was checked during the 7 days of incubation. In several studies, this method was used to determine the structural changes in proteins during glycation such as protein instability and α-helix modifications [33,34,35,36]. In Figure 5, the CD spectra of HSA and gHSA with or without ASX and ASX-metal ions incubated at different times are reported. The CD spectra of albumin protein exhibits two negative bands at 209 and 222 nm, characteristic of the alpha-helix structure [37]. In Figure 6, the percentage of protein secondary structure for all samples analyzed at D-0 and D-7 are shown. The blue and pink bars indicate the α-helical and random coils content, respectively.

A lower delta epsilon value, and therefore an increase in alpha-helix content was observed for gHSA (red line) compared with native HSA (black line) at D-0 (Figure 5). Furthermore, increasing the incubation time, the α-helix percentage in HSA increased from 48% (D-0) to 55.4% (D-7), close to the α-helix content in gHSA, which is 57% (D-0) (Figure 6). The CD data, recorded at D-1, D-3 and D-5 are reported in the Supplementary Materials (Figures S2 and S3). These findings are in agreement with the results reported by Sattarahmady et al. [36] and Raghav et al. [38], where α-helix in gHSA was higher than HSA, with a decrease in β-sheet conformation, an increase in mean residual ellipticity, and random coil structures.

The complexation of proteins with ASX and ASX-metal ions led to a lowering in the α-helical content, caused by the new bonds. Furthermore, depending on the molar ratio and the concentration of ASX and ASX-metal ions, an increase in protein stability was observed. The best result for ASX was for gHSA-ASX 20 μM (sample 5), where a decrease of 1.7% of α-helix from D-0 to D-7 was observed (Figure 6). For the complex ASX-Cu, the best molar ratio was 3:1. A variation in α-helical content of 1.8 and 1.3% for gHSA-ASXCu (3:1) (sample 15) and HSA-ASXCu (3:1) (sample 16), respectively, was found. For ASX-Zn, the 1:2 and 1:1 were the best ratios to maintain α-helical content from gHSA to normal HSA values. In general, the addition of ASX and ASX-metal ions had a stability impact, especially on glycated albumin, by maintaining the secondary protein structure close to the initial conditions such as normal albumin. The stabilization of albumin proteins, especially for gHSA, was also confirmed by an increase in random coils in all samples (Figure 6 pink bar). Yang et al. [39] showed that the addition of AGuIX^®^, which is a nanoparticle-based cancer therapy drug, had also the same effect, namely the increase in others/random coils in HSA after drug administration increased the stability of human serum albumin protein. It can be concluded that ASX and ASX-metal ions have a positive impact on the stabilization of albumin proteins, especially on gHSA. These results are in line with molecular dynamic simulation and density functional theory carried out in our previous studies [9,17], where ASX-metal ions prevented protein unfolding better than native state ASX.

### 2.5. Viscosity, Particle Size and Zeta Potential Analysis

**Figure 7:**
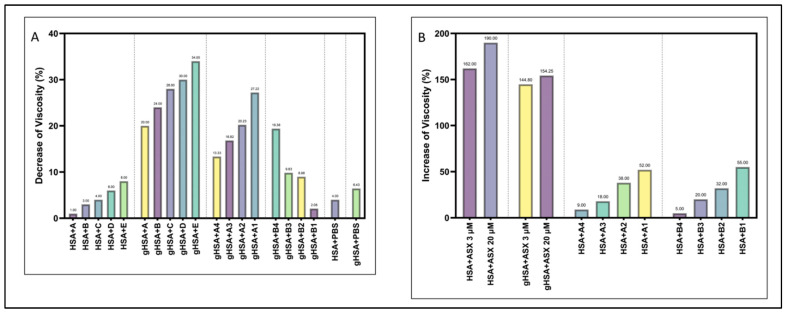
Viscosity result of HSA/gHSA + ASX/ASX-metal ions. Decrease in viscosity (**A**) and increase in viscosity (**B**). The experiments were carried out for several samples, ASX at various concentrations: A = 2 × 10^−10^ mM, B = 1 × 10^−10^ mM, C = 8 × 10^−10^ mM, D = 6 × 10^−11^ mM, E = 4 × 10^−11^ mM. ASX-metal ion complex where ASX-Cu with ratio (3:1) is divided into A1 (100% complex (6 mL ASX-Cu (3:1)), A2 (75% complex (4.5 mL ASX-Cu (3:1) + 1.5 mL PBS)), A3 (50% complex (3 mL ASX-Cu (3:1) + 3 mL PBS), A4 (25% complex (1.5 mL ASX-Cu (3:1) + 4.5 mL PBS)) and A5 (6 mL PBS). ASX-Zn (20:20) is divided into B1 (100% complex (6 mL ASX-Zn (20:20)), B2 (75% complex (4.5 mL ASX-Zn (20:20) + 1.5 mL PBS)), B3 (50% complex (3 mL ASX-Zn (20:20) + 3 mL PBS), B4 (25% complex (1.5 mL ASX-Zn (20:20) + 4.5 mL PBS)) and B5 (6 mL PBS). Total volume was 12 mL (6 mL proteins (gHSA or HSA) + 6 mL compounds) and incubated for 7 days, 37 °C and 110 rpm.

Many complications in DMT2 patients are related to increases in the blood viscosity. To allow albumin to work properly, viscosity should be decreased, especially for gHSA [40]. In this context, viscosity analysis was performed for HSA and gHSA in the presence of ASX and ASX-metal ions. The viscosity analysis showed a viscosity reduction in gHSA due to the presence of ASX and ASX complex with Cu^2+^ and Zn^2+^ (Figure 7). An important role was played by the concentration of ASX, because ASX in little concentration reduced the viscosity of HSA and gHSA of 34% and 8%, respectively, while higher concentrations (3 and 20 μM) affected viscosity of gHSA, increasing to 144.80% and 154.25%. Adding metal ions such as Cu^2+^ and Zn^2+^, the viscosity of gHSA decreased while the viscosity value for HSA increased. ASX-Cu^2+^ (3:1) complexed with gHSA for 7 days showed a decrease in viscosity of 27.22%. Meanwhile, for ASX-Zn^2+^ (20 µM:20 µM) as much as 25% of complex compound (1.5 mL (ASX-Zn^2+^) + 4.5 mL PBS) was able to reduce gHSA viscosity by 19.38%. In conclusion, ASX at low concentration (6 × 10^−11^ mM) could be used to reduce the viscosity of HSA; but considering that for DMT2 patients, a decrease in the viscosity of gHSA is essential, the use of ASX-metal ions could be relevant.

The particle size analysis (Table 1) shows the increase in size for gHSA and HSA proteins complexed with astaxanthin, due to the formation of aggregates in the form of fibrils [41] A higher zeta potential value in gHSA (−14.6 mV) has been found compared to normal HSA (−10.0 mV). This happened because arginine and lysine residues were major sites for glycation. When this modification occurred, it was followed by an increase in negative charge and a decrease in the isoelectric point of the protein. A higher hydrophobicity for the glycated protein due to protein conformational changes, including partial unfolding, will result [42]. Then, when HSA and gHSA were complexed with ASX, the zeta potential value for the two proteins was smaller, namely HSA-ASX (−2.88 mV) and gHSA-ASX (−4.82 mV). However, when compared with the ASX-metal ion complexes, the ability of ASX in energy stabilization was better, especially for gHSA, where for ASX-Cu^2+^ (−8.58 mV) and ASX-Zn^2+^ (−10.1 mV).

The main objective of zeta potential analysis is to detect a lower zeta potential while retaining a good conductivity value in the chemical complex of ASX with gHSA. The decrease in zeta potential value was also followed by an increase in conductivity where gHSA + ASX (5.58 mS/cm), gHSA + ASX (5.11 mS/cm) and gHSA + ASX (3.35 mS/cm) became 7.61 mS/cm (gHSA + ASX-Zn) and 6.58 mS/cm (gHSA + ASX-Cu). This shows that metal ions-ASX complex is able to provide a better protective effect than native ASX on the protein albumin structure. This is supported by the study of Hang Ma et al. [42] where gBSA before treatment had a zeta potential value of −106.3 mV and when given PGG (gallotannin) decreased to −30.9 mV. The data showed decreased zeta potential for all ASX and ASX-metal ions + HSA/gHSA complexes. However, when compared to ASX, ASX-metal ions perform much better in terms of maintaining conductivity in the sample, so we can conclude from zeta potential and conductivity data that the addition of metal ions to ASX can improve astaxanthin’s ability to carry out energy stabilization in the HSA or gHSA complexes.

### 2.6. Molecular Docking

**Figure 8:**
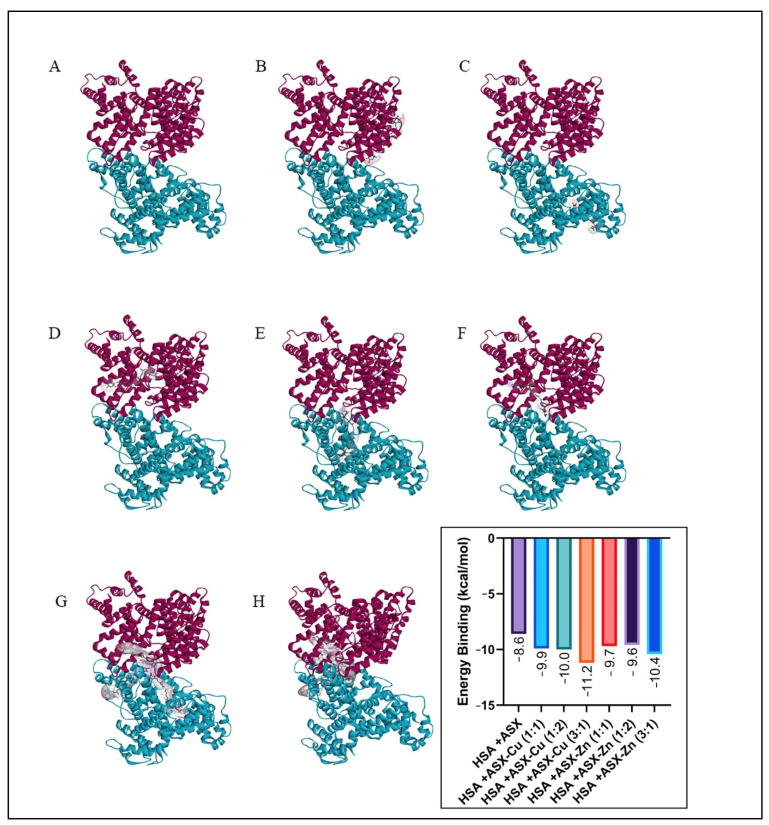
Docking result for ASX/ASX-metal ions with HSA: (**A**) HSA, (**B**) HSA+ASX, (**C**) HSA+ASX-Cu (1:1), (**D**) HSA+ASX-Cu (1:2), (**E**) HSA+ASX-Zn (1:1), (**F**) HSA+ASX-Zn (1:2), (**G**) HSA+ASX-Cu (3:1) and (**H**) HSA+ASX-Zn (3:1).

**Figure 9:**
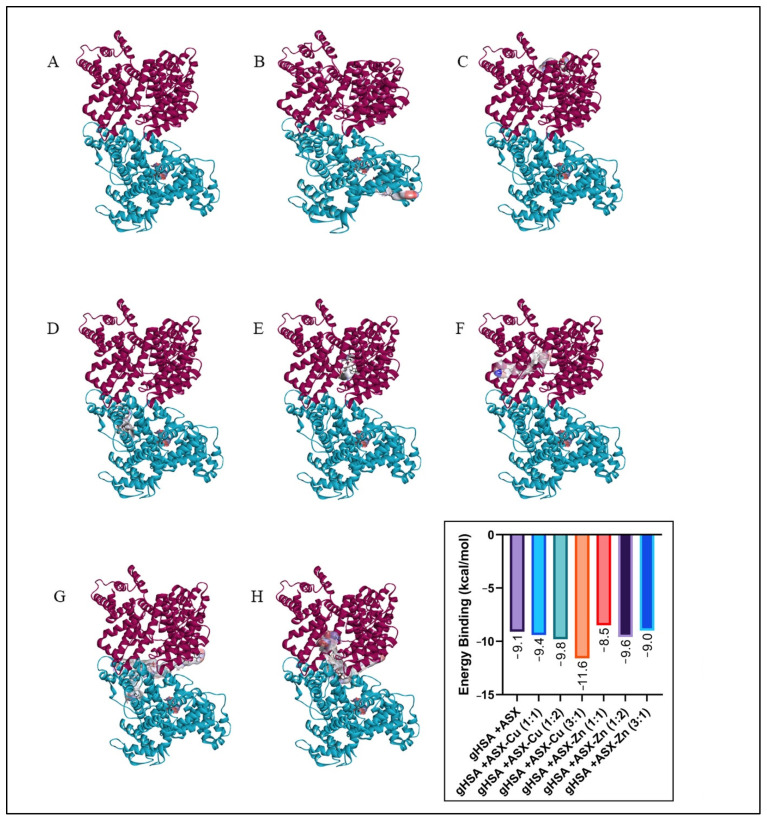
Docking results for ASX/ASX-metal ions with HSA (**A**) gHSA, (**B**) gHSA+ASX, (**C**) gHSA+ASX-Cu (1:1), (**D**) gHSA+ASX-Cu (1:2), (**E**) gHSA+ASX-Zn (1:1), (**F**) gHSA+ASX-Zn (1:2), (**G**) gHSA+ASX-Cu (3:1) and (**H**) gHSA+ASX-Zn (3:1).

The glycated albumin model used in this study has hexose (206) as a glycation agent, which is different from previously used glucose (5793) [9,17]. The results for the molecular docking (Figure 8 and Figure 9) showed a slight difference in the binding site of ASX and ASX-metal ion complexes towards albumin when compared with previous studies [17]. When glucose is used as glycation agent, ASX and ASX-metal ions were uniformly bound to site B on gHSA. In all cases, the ASX and ASX-metal ions binding was not competitive with amino acid residues at the glucose binding site. Whereas both ASX and ASX-metal ions will be bound to the A site [9,17].

**Figure 10:**
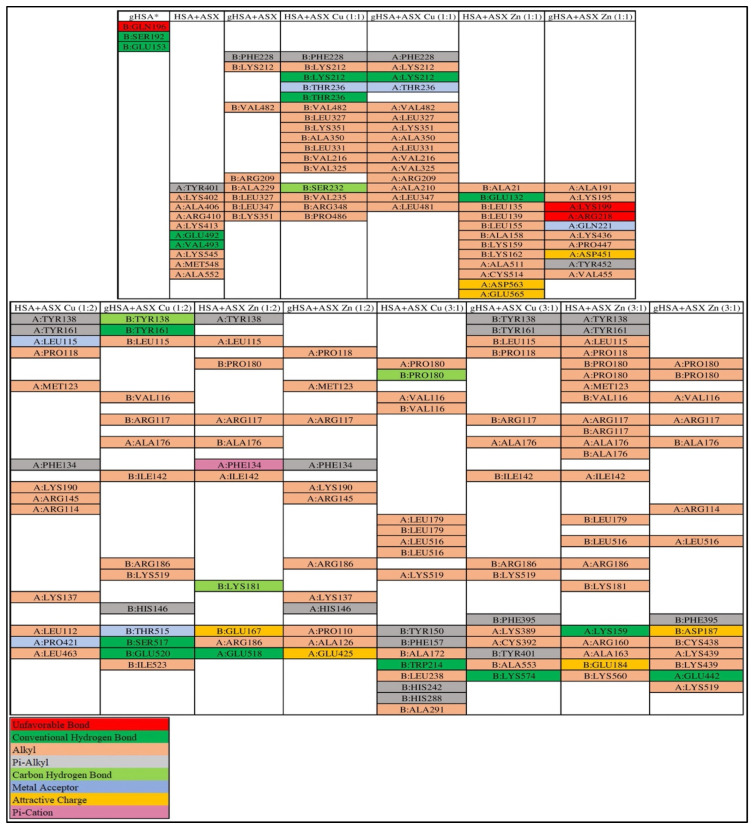
Binding sites for ASX/ASX-metal ions with HSA and gHSA (*HSA complex with glycation agent).

Then, the consistency of the binding site (Figure 10) then only occurred at a few concentrations. This is because different glycation agents also cause different folding effects. Binding location for glycation using hexose is still at site B of the albumin HSA protein, namely B:GLN196 (unfavorable bond), B:SER192 (conventional hydrogen bond) and B:GLU153 (conventional hydrogen bond). While for glucose as a glycation agent, the unfavorable bond is at residue B:GLN459 and conventional hydrogen bond at B:SER193, B:ARG145 and B:ASP108 [9,17].

From binding energy, we can conclude that ASX-metal ions are still better than ASX when binding into HSA, HSA+ASX (−8.6 kcal/mol), meanwhile HSA+ASX-Cu (3:1) (−11.2 kcal/mol) and HSA+ASX-Zn (3:1) is −10.4 kcal/mol. These findings support all the data for UV-Vis, CD-spectra, antioxidant activity by EPR, viscosity as well as zeta potential. Both ASX and ASX-metal ions interact more strongly with gHSA than HSA complex. Where in gHSA, ASX has a binding energy of −9.1 kcal/mol, while in HSA, it is −8.6 kcal/mol. Likewise, the addition of metal ions Cu^2+^ and Zn^2+^ can increase the binding energy of ASX in gHSA protein.

However, in ASX-Zn (1:1), the bond energy was found to be lower than ASX, when viewed from the ASX-Zn data with a 1:1 ratio with a concentration of 1 μM:1 μM, it is still able to maintain the secondary structure of HSA and gHSA until the 7th day of incubation. For concentration (20 μM:20 μM) (1:1), the CD-spectra pattern for HSA and gHSA is still the same, but drastic changes occurred in the secondary structure. This is because there are unfavorable bonds in the gHSA + ASX-Zn (1:1) complex in the amino acid residues A:ARG218 and A:LYS199 and the concentration used is most likely higher than the protein concentration (1 μM). Data docking also supports scavenging activity of astaxanthin where ASX-Zn (1:2) which has the lowest binding energy towards gHSA (−9.6 kcal/mol) and better scavenging activity 55.2% compared to ASX alone. The same trend is also shown by ASX-Cu (3:1) with the lowest binding energy of −11.6 kcal/mol and the largest scavenging activity of 62.9% (Figure 3 and Figure 9). The lower the binding energy, the better the interaction with the target protein.

## 3. Materials and Methods

Astaxanthin (SML0982, CAS No 472-61-7), human serum albumin (HSA) and glycated human serum albumin (gHSA), CuCl_2_·2H_2_O, ZnCl_2_, absolute ethanol, phosphate-buffered saline (PBS) were obtained from Sigma–Aldrich.

### 3.1. ASX-Transition Metal Ion Complexes

Astaxanthin stock solution was obtained by dissolving 10 mg in 100 mL ethanol (0.167 mM). After, solutions with different astaxanthin concentrations, as native (6 × 10^−11^ mM) and as metal complexes with Cu^2+^ (1 μM and 3 μM) and Zn^2+^ (1 μM, 3 μM and 20 μM), were prepared. These concentrations are based on the properties, previously determined, for the preparation of ASX and ASX-metal ion complexes [18]. The Cu^2+^ stock solution was prepared with CuCl_2_·2H_2_O (6.814 mg) in 20 mL aqueous solution and then used as a working solution with a concentration of 1 and 2 μM. Zn^2+^ was prepared by dissolving ZnCl_2_ (54.52 mg) in 20 mL of aqueous solution, then diluted into working solutions with concentrations of 1, 2 and 20 μM. For the complex formation, the following conditions were used: stirrer time (5 min for ASX-Cu and 15 min for ASX-Zn), temperature (351 K) and complex molar ratio of (astaxanthin:metal ions) both for Cu^2+^ and Zn^2+^ (1:1, 1:2 and 3:1). Temperature control was carried out on a water bath placed on top of magnetic stirrers (Cimarec Thermom SP-88850105) at a speed of 110 rpm [17,18].

### 3.2. ASX-HSA and ASX-gHSA Complexes

HSA (33.2 mg) and gHSA (16.6 mg) were dissolved in 100 and 50 mL PBS (5 µM), respectively, and diluted to 1 µM concentration. ASX–protein complex was made by incubating samples with temperature conditioning (280 K) in a dark environment on a shaking incubator at a speed of 110 rpm for 7 days. Sample codes are 1 (HSA 1 μM), 2 (gHSA 1 μM), 3 (gHSA-ASX 3 μM), 4 (HSA-ASX 3 μM), 5 (gHSA-ASX 20 μM), 6 (HSA-ASX 20 μM), 7 (gHSA-ASX 6 × 10^−11^ mM), 8 (HSA-ASX 6 × 10^−11^ mM), 9 (gHSA-ASX 1 μM), 10 (HSA-ASX 1 μM), 11 (gHSA-ASXCu (1:1) μM), 12 (HSA-ASXCu (1:1) μM), 13 (gHSA-ASXCu (1:2) μM), 14 (HSA-ASXCu (1:2) μM), 15 (gHSA-ASXCu (3:1) μM), 16 (HSA-ASXCu (3:1) μM), 17 (gHSA-ASXZn (1:1) μM), 18 (HSA-ASXZn (1:1) μM), 19 (gHSA-ASXZn (1:2) μM), 20 (HSA-ASXZn (1:2) μM), 21 (gHSA-ASXZn (3:1) μM), 22 (HSA-ASXZn (3:1) μM), 23 (gHSA-ASXZn (20:20) μM) and 24 (HSA-ASXZn (20:20) μM).

### 3.3. UV-Vis Spectrophotometric Characterization

The UV-Vis experiments were performed using a UV-Vis Spectrophotometer Lambda 900/Perkin Elmer Instruments (Norwalk, CT, USA). Each sample of ASX was reviewed for UV-Vis spectrum profile with a wavelength of 190–790 nm meanwhile for HSA, gHSA and complexes in 190–690 nm range, using a survey mode (accuracy of 5 nm). The spectrophotometer calibration was performed using absolute ethanol and PBS for ASX and ASX–protein complexes, respectively.

### 3.4. Viscosity Measurement

Measurements were performed by using the simple capillary tube viscometer method at Klinik Bromo, Malang. All the samples were obtained in sets of three and averaged. A 2 mL reservoir was located on the upper section of the viscometer. It was filled vertically with fluid sample to the reservoir’s upper line, and the free flow time of the sample to the reservoir’s lower line was measured in seconds (s). The value obtained by comparing the free flow time of a sample to the free flow time of distilled water, which is 1, is referred to as “relative viscosity”. The viscometer was used in the same vertical position and without direct sunlight or airflow under the designated constant laboratory conditions. To make the statistical and graphic estimates more accurate, free flow time instead of relative viscosity value was used [40].

### 3.5. CD-Spectroscopy Analysis

The spectropolarimeter used was a J-815 model (Jasco). Samples were placed in quartz cells with a size of 0.1 cm. The spectral range, 190–250 nm, was used. The temperature was 298 K, with a response time of 2 s and a scanning speed of 20 nm/min in continuous scanning mode. The sensitivity was standard (100 mdeg), and the data pitch and band width were 0.1 nm and 1 nm, respectively. Data were accumulated three times. Time dependent analysis was divided into 5 days: day 0, 1, 3, 5 and 7 (incubation time of samples). For the analysis of the CD data, the spectrum of the PBS buffer was subtracted for all complexes. PBS was used as protein solvent. The data were then computationally processed using a web server for secondary structure analysis, specifically BeStSel (Beta Structure Selection) in (bestsel.elte.hu/index.php) [43].

### 3.6. Particle Size and Zeta Potential Analysis

The zeta potential and particle size analysis of each sample were measured with a Zetasizer Nano-ZS90 (Malvern, Worcestershire, UK) using laser doppler micro-electrophoresis technique. For this purpose, 17 samples, each in triplicate, were prepared in PBS buffer, pH 7.4, and incubated in the dark at 310 K for 7 days prior to analyses: HSA (1 μM), gHSA (1 μM), ASX (6 × 10^−11^ mM), HSA + ASX, gHSA + ASX, ASX-Cu^2+^, ASX-Zn^2+^, ASX 3 μM, ASX 20 μM, (ASX-Cu^2+^) + HSA, (ASX-Cu^2+^) + gHSA, (ASX-Zn^2+^) + HSA, (ASX-Zn^2+^) + gHSA, (ASX 3 μM) + HSA, (ASX 3 μM) + gHSA, (ASX 20 μM) + HSA, and (ASX 20 μM) + gHSA. Selection of ASX sample concentration (6 × 10^−11^ mM) was based on viscosity results, which showed high stability in performing functions to reduce blood viscosity in gHSA. Meanwhile, based on our previous research, the concentration of astaxanthin with metal ions was chosen as follows: ASX-Cu (3:1), T = 351 K, 5 min and ASX-Zn (1:1), T = 351 K, 15 min [18]. The operating temperature was set at 298 K and 100 consecutive runs were collected for each sample.

### 3.7. Electron Paramagnetic Resonance Spectroscopy (EPR)

EPR measurements were performed with a Bruker E580 Elexsys Series spectrometer (Bruker Biospin GmbH, Rheinstetten, Germany), operating at X- band (ν = 9 GHz) and equipped with a Bruker high sensitivity ER4122SHQE cavity.

For antioxidant analysis, the 2,2-diphenyl-picrylhydrazyl (DPPH) assay was chosen. DPPH (0.1 mM) reacted with both ASX and ASX-metal ions with a final concentration of 0.25 µM. The incubation time was 30 min and experiments were performed in ethanol at T = 298 K. After the spectra acquisition of DPPH radical signal in absence and presence of ASX and ASX-metal ions, the double integral of each spectrum was calculated and the scavenger activity percentage was determined using the following formula:

$$\mathrm{Scavenger}\;\mathrm{effect}\;(\%)=\frac{A_{0}-A_{a}}{A_{a}}\times 100$$

where *A_0_* represents the double integral of the DPPH radical without the addition of the antioxidant, *A_a_* corresponds to the double integral of the DPPH radical after the addition of antioxidant.

Meanwhile, because Cu^2+^ is a paramagnetic metal ion and Zn^2+^ is not, the EPR spectra have been recorded to check the complex formation between ASX and Cu^2+^ metal ion. The samples were prepared in a 1 mm ID quartz capillary tube and then were placed inside standard Suprasil EPR tubes. The low temperature spectra were recorded using the Bruker ER 4111VT variable temperature unit. All samples were analyzed at low temperature (130 K) using liquid nitrogen.

### 3.8. Molecular Electrostatic Potential (MEP) Analysis

The ASX and ASX-metal ion complexes were created in the Avogadro program by input of the isomeric SMILES of ASX and then adding Zn^2+^ and Cu^2+^. A setup force field (UFF), a 500-step method, and an algorithm (Steepest Descent) to optimize the geometry were also used. All computations were carried out using the TD-DFT software Gaussian09 and GaussView 6.0 [44,45,46]. For frequency analysis and optimization, the Ground State approach was utilized, with the LANL2DZ basis set [47,48,49,50] and B3LYP density functional [51,52,53]. The LANL2DZ basis set was chosen because it fits the requirements for TD-DFT calculations on the interaction of metal ions such as cadmium and zinc [54,55,56,57,58,59].

### 3.9. Molecular Docking

PubChem (www.pubchem.ncbi.nlm.nih.gov accessed on 1 July 2021) and PDB (www.rscb.org/pdb accessed on 1 July 2021) databases were used for data mining. The compounds used were ASX (5,281,224), hexose (206), Zn^2+^ (32,051) and Cu^2+^ (27,099). The ASX-metal ion complexes were divided into three, which were ASX-M^2+^, ASX-2M^2+^ and 3ASX-M^2+^. Meanwhile, HSA with PDB ID (4K2C). The ASX and ASX-metal ions were prepared in the Open Babel software by changing the format (.sdf) to (.pdbqt), after which the HSA protein was cleaned of ligand and water using the Discovery Studio 2016 Client and stored in (.pdb) format. AutoDock Vina, that is included in the PyRx 0.8 package, was used for docking. Autodock Vina was selected as it adopts more accurate binding poses than Autodock 4 which are in line with the research objective, namely supporting the results for the wet lab research data with in silico data [60]. All docking operations with dimensions: X (90.1439), Y (109.1736), and Z (79.3440) are covered by the Vina search box, while the center of the box uses X (11.5350), Y (−23.336), and Z (5.6978) [6,9,17].

## 4. Conclusions

For the first time ever, EPR data were obtained reporting the complexation of ASX with metal ion (Cu^2+^). Its relationship with HSA and gHSA, as well as the ability of ASX/ASX-metal ions (Cu^2+^ and Zn^2+^) as antioxidants, showed by 2,2-diphenyl-picrylhydrazyl (DPPH) assay and MEP analysis, was reported. With the addition of ASX/ASX-metal ions, the ability of gHSA (an essential marker in T2DM patients) as a transporter and endogenous scavenger improved protein rheology, decreasing viscosity and zeta potential close to normal albumin values. CD spectroscopy also confirmed that ASX and ASX-metal ion complexes prevent damage to gHSA by maintaining the stability of the secondary structures from D-0 until D-7. It was also observed that ASX-metal ions improved the stability of gHSA more than ASX alone. The docking data confirm that the best binding energy is for ASX-Cu^2+^ (3:1) and ASX-Zn^2+^ (1:2); it is also in line with the antioxidant data and CD spectroscopy, which show that the above ratios are the best in maintaining the stability of albumin protein, both native and glycated. Overall, adding metal ions to astaxanthin increase astaxanthin’s ability as an antioxidant and help to maintain the stability of HSA and gHSA in carrying out its functions.

## Figures and Tables

**Table 1:** Particle size, zeta potential and conductivity data for ASX and ASX-metal ions + HSA/gHSA complexes.

Samples	Z-Average (d.nm)	Polydispersity Index	Zeta Potential (mV)	Conductivity (mS/cm)
HSA (1 μM)	199.4	0.248	−10.0	15.9
gHSA (1 μM)	191.1	0.674	−14.6	16.0
ASX (6 × 10^−11^ mM)	390.5	0.686	−0.404	0.002
HAS + ASX	9331	0.354	−2.88	4.37
gHSA + ASX	3969	0.377	−4.82	5.58
ASX-Cu^2+^	939.1	0.586	2.32	0.134
ASX-Zn^2+^	889.8	0.762	−2.70	0.953
ASX 3 μM	815.8	0.463	−3.30	0.0184
ASX 20 μM	971.1	0.539	−1.22	0.00683
(ASX-Cu^2+^) + HSA	6458	0.638	−6.93	6.19
(ASX-Cu^2+^) + gHSA	6418	0.350	−8.58	6.58
(ASX-Zn^2+^) + HSA	937.0	0.640	−9.23	8.09
(ASX-Zn^2+^) + gHSA	990.2	0.661	−10.1	7.61
(ASX 3 μM) + HSA	8995	0.492	−5.09	5.18
(ASX 3 μM) + gHSA	7209	0.601	−4.25	5.11
(ASX 20 μM) + HSA	7136	0.590	−2.45	4.48
(ASX 20 μM) + gHSA	2673	0.449	−2.36	3.35

## Data Availability

Not Applicable.

## Supplementary Materials

The following supporting information can be downloaded at: https://www.mdpi.com/article/10.3390/ijms23094771/s1.
