# Supplementary material for: Quantification and Improvement of the Dynamics of Human Serum Albumin and Glycated Human Serum Albumin with Astaxanthin/Astaxanthin-Metal Ion Complexes: Physico-Chemical and Computational Approaches
Source: Int J Mol Sci. 2022 Apr 26;23(9):4771. doi: 10.3390/ijms23094771 (PMC9104927; doi:10.3390/ijms23094771)
Supplement: Supplementary file 1 [file ijms-23-04771-s001.zip › ijms-1689704-supplementary.pdf]

Supplementary Materials

Quantification and Improvement of the Dynamics of Human Serum Albumin and Glycated Human Serum Albumin with Astaxanthin/Astaxanthin-Metal Ion Complexes: Physico–Chemical and Computational Approaches

Syahputra Wibowo[1*], Jessica Costa[2,3], Maria Camilla Baratto[2,3], Rebecca Pogni[2,3*], Sri Widyarti[1], Akhmad Sabarudin[4], Koichi Matsuo[5], Sutiman Bambang Sumitro[1*]

[1]Department of Biology, Faculty of Mathematics and Natural Sciences, Brawijaya UniversityJl. Veteran, Malang 65145, East Java, Indo
[2]University of Siena, Department of Biotechnology, Chemistry and Pharmacy, Via A. Moro 2, 53100, Siena, Italy
[3]CSGI,Consorzio per lo Sviluppo dei Sistemi a Grande Interfase, Via della Lastruccia 3, Sesto Fiorentino, 50019, Italy
[4]Department of Chemistry, Faculty of Mathematics and Natural Sciences, BrawijayaUniversity, Jl. Veteran, Malang 65145, East Java, Indonesia.
[5]Hiroshima Synchrotron Radiation Center, Hiroshima University, Higashi-Hiroshima 739-0046, Japan
*Correspondence: wibowo@student.ub.ac.id; Tel.: +6281238281075 (SW (Syahputra Wibowo); RP (Rebecca Pogni, rebecca.pogni@unisi.it); SBS (Sutiman Bambang Sumitro, sutiman@ub.ac.id)

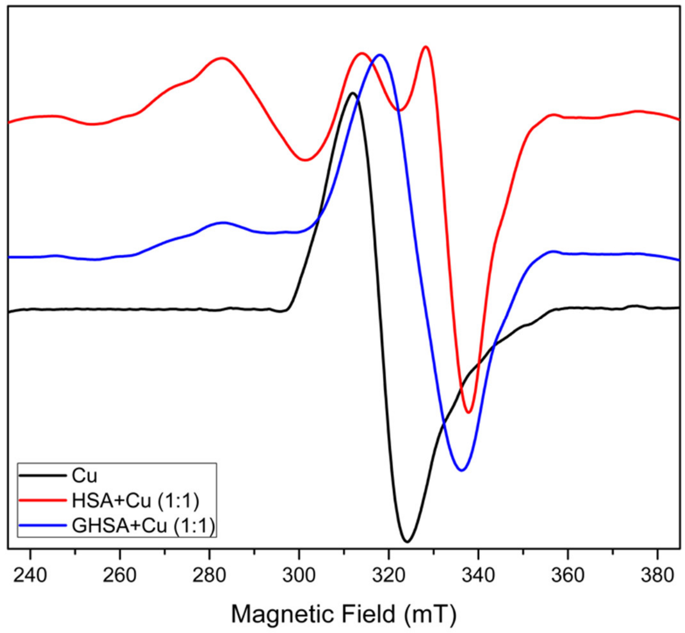

Figure S1. 150K X-band EPR spectra of: free Cu (black line), HSA+Cu (1:1 (red line), gHSA+Cu (1:1) (blue line). *Experimental conditions: ν=9.67GHz, 21mW microwave power and 0.5mT modulation amplitude.*

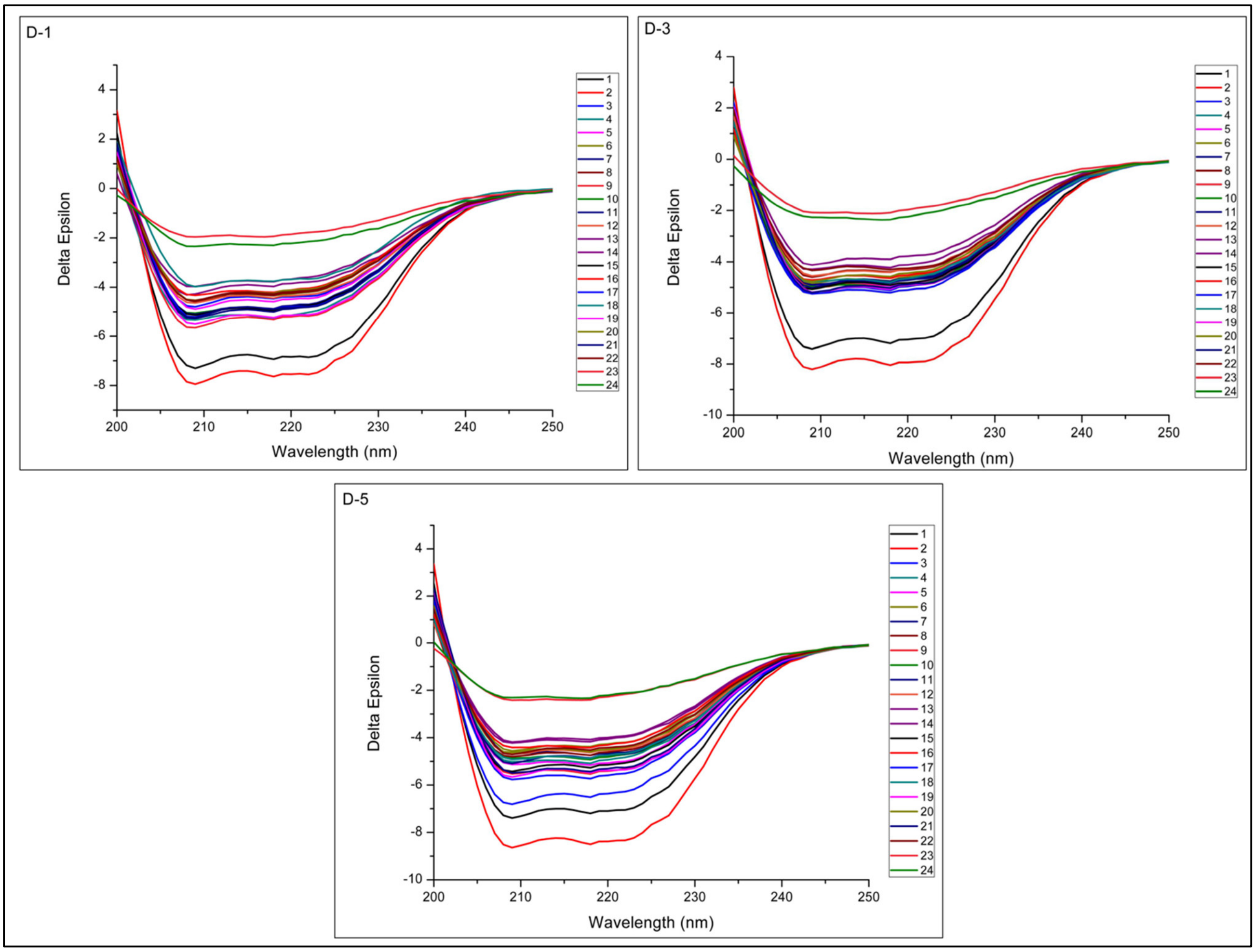

Figure S2. CD spectra of all samples (the numbers refer to the different samples reported in Materials and method) after incubation at T = 310K recorded at Day 1 (D-1), Day 3 (D-3), and Day 5 (D-5).

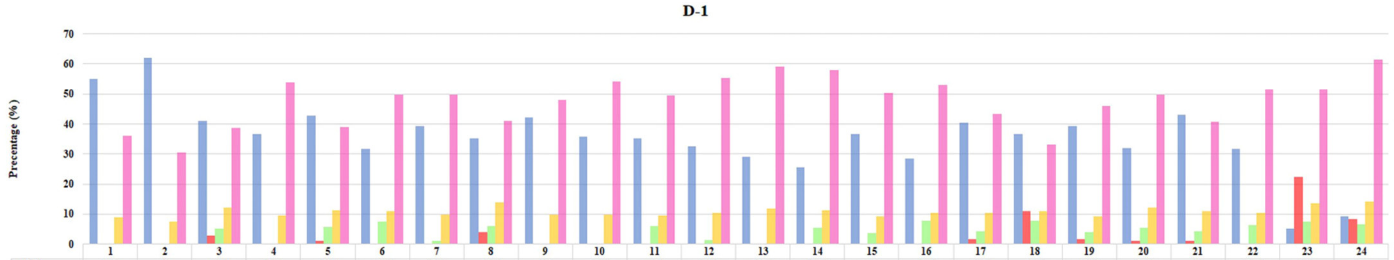

	1	2	3	4	5	6	7	8	9	10	11	12	13	14	15	16	17	18	19	20	21	22	23	24
Helix:	54.9	62	41.1	36.8	42.8	31.8	39.4	35.1	42.3	35.9	35.2	32.7	29.2	25.6	36.7	28.6	40.4	36.6	39.3	31.9	43	31.7	5.2	9.3
Antiparallel:	0	0	2.9	0	1.1	0	0	3.9	0	0	0	0	0	0	0	0	1.6	11.1	1.6	1	1	0	22.3	8.4
Parallel:	0	0	5.1	0	5.8	7.5	1.2	6.1	0	0.2	5.9	1.5	0	5.4	3.7	7.9	4.2	7.9	3.9	5.4	4.2	6.4	7.5	6.5
Turn:	9	7.6	12.3	9.4	11.3	10.9	9.7	13.9	9.8	9.8	9.4	10.5	11.8	11.3	9.1	10.4	10.4	11.1	9.2	12.1	11.1	10.5	13.5	14.3
Others:	36.1	30.4	38.6	53.8	39	49.7	49.7	41	47.9	54.1	49.4	55.3	59	57.8	50.5	53	43.4	33.3	45.9	49.7	40.7	51.4	51.5	61.4

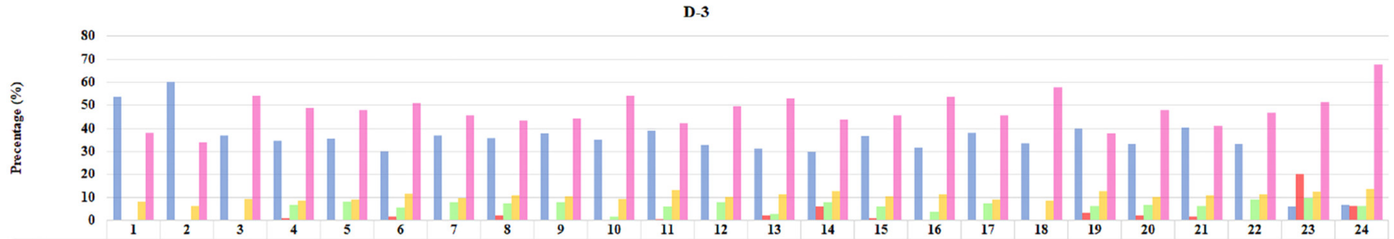

	1	2	3	4	5	6	7	8	9	10	11	12	13	14	15	16	17	18	19	20	21	22	23	24
Helix:	53.8	60	36.8	34.8	35.3	30.2	36.8	35.9	37.6	34.9	38.8	32.7	31.3	29.8	36.6	31.5	38.3	33.6	40	33.2	40.5	33.1	5.9	6.5
Antiparallel:	0	0	0	1.1	0	1.7	0	2.1	0	0	0.1	0	2	5.9	1.1	0	0	0	3.2	2.2	1.6	0	20.4	6.2
Parallel:	0	0	0	6.5	8	5.4	7.6	7.5	7.8	1.8	5.7	7.9	2.9	7.6	5.9	3.5	7.3	0	6.4	6.6	6.2	9	9.7	6.2
Turn:	8	6.1	9.4	8.7	8.8	11.6	9.8	10.8	10.3	9.2	13	9.9	11	12.7	10.6	11.3	8.8	8.6	12.8	10.2	10.7	11	12.4	13.3
Others:	38.2	33.9	53.9	48.9	48	51	45.8	43.6	44.3	54.1	42.3	49.5	52.9	44	45.9	53.7	45.6	57.9	37.6	47.9	41.1	46.8	51.5	67.7

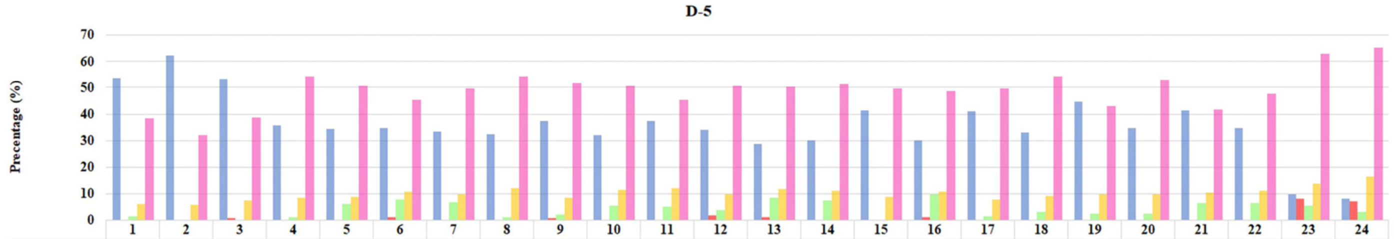

	1	2	3	4	5	6	7	8	9	10	11	12	13	14	15	16	17	18	19	20	21	22	23	24
Helix:	53.6	62.3	53.2	35.8	34.4	34.7	33.4	32.5	37.4	32.1	37.3	34.1	28.6	30.2	41.3	29.9	40.9	33.2	44.6	34.7	41.5	34.6	9.9	8.2
Antiparallel:	0	0	0.7	0	0	1.3	0	0	0.7	0	0	1.8	1.1	0	0	1	0	0	0	0	0	0	8	7
Parallel:	1.6	0	0	1.3	6.3	7.8	6.9	1.1	2	5.6	5.3	3.7	8.4	7.4	0	9.7	1.6	3.3	2.6	2.5	6.4	6.6	5.5	3.2
Turn:	6.3	5.7	7.4	8.5	8.7	10.8	9.9	12	8.4	11.5	12.2	9.9	11.7	11	8.9	10.8	7.9	9.2	9.9	9.9	10.4	11.1	13.9	16.4
Others:	38.4	32.1	38.8	54.4	50.6	45.3	49.8	54.4	51.5	50.8	45.3	50.5	50.3	51.3	49.7	48.7	49.5	54.3	42.9	53	41.8	47.7	62.8	65.2

Figure S3. Percentage of secondary structures calculated using the BestSel program: **1** (HSA 1 µM), **2** (gHSA 1 µM), **3** (gHSA-ASX 3 µM), **4** (HSA-ASX 3 µM), **5** (gHSA-ASX 20 µM), **6** (HSA-ASX 20 µM), **7** (gHSA-ASX 6 x10^{-11} mM), **8** (HSA-ASX 6 x10^{-11} mM), **9** (gHSA-ASX 1 µM), **10** (HSA-ASX 1 µM), **11** (gHSA-ASXCu (1:1) µM), **12** (HSA-ASXCu (1:1) µM), **13** (gHSA-ASXCu (1:2) µM), **14** (HSA-ASXCu (1:2) µM), **15** (gHSA-ASXCu (3:1) µM), **16** (HSA-ASXCu (3:1) µM), **17** (gHSA-ASXZn (1:1) µM), **18** (HSA-ASXZn (1:1) µM), **19** (gHSA-ASXZn (1:2) µM), **20** (HSA-ASXZn (1:2) µM), **21** (gHSA-ASXZn (3:1) µM), **22** (HSA-ASXZn (3:1) µM), **23** (gHSA-ASXZn (20:20) µM) and **24** (HSA-ASXZn (20:20) µM).
